# Case Report: Human Recombinant Growth Hormone Therapy in a DSH Cat Presented With Dwarfism

**DOI:** 10.3389/fvets.2021.773355

**Published:** 2021-11-22

**Authors:** Martina Načeradská, Kateřina Návojová Horáčková, Michaela Fridrichová

**Affiliations:** ^1^Department of Veterinary Sciences, Faculty of Agrobiology, Food and Natural Resources, Czech University of Life Sciences in Prague, Prague, Czechia; ^2^Veterinární ordinace MVDr. Martiny Načeradské, Prague, Czechia; ^3^Veterinární ordinace U stříbrné kočičky, Prague, Czechia; ^4^Department of Inorganic Chemistry, Faculty of Science, Charles University, Prague, Czechia

**Keywords:** dwarfism, human growth hormone, cat, hyposomatotropism, IGF-I

## Abstract

A 6-month-old kitten, male, domestic shorthair cat was presented with dwarfism, ocular and nasal discharge, and *Ascaris* infestation. Congenital hyposomatotropism was diagnosed on the basis of serum level of insulin-like growth factor-1 (IGF-I). The cat was treated with human recombinant growth hormone for 9 weeks. After that, his liver enzymes became elevated, and the therapy was discontinued. His IGF-I levels were normal at the end of the therapy. Normal IGF-I was present 3 months after discontinuation of therapy with human recombinant growth hormone and even half a year after the discontinuation. All other comorbidities were addressed with the therapy. The cat is now the size of normal cats, living with the first author.

## Introduction

There are several causes of dwarfism, “failure to thrive” in cats. Failure to grow can be caused by non-endocrine and endocrine disorders. Non-endocrine disorders are much more common and comprise malnutrition, dysfunction of nearly any organ in the body, and any chronic disease. Also included is constitutional dwarfism, which is a normal growth variation resulting in small stature in an otherwise healthy individual. Signs and symptoms seen at presentation can help direct further investigations. Presence of tachycardia or a heart murmur may be a sign of a cardiac abnormality. Regurgitation or vomiting may be the consequence of swallowing disorders such as megaesophagus or vascular ring anomaly. Diarrhea may suggest maldigestion or malabsorption caused by pancreatic disorders or intestinal parasites. Polydipsia and polyuria can be present in renal and hepatic disorders. When mental dullness is present, portosystemic shunt or central nervous system disorders should be suspected (1, 2).

Systemic diseases that can cause abnormal skeletal development in cats include nutritional imbalances such as nutritional secondary hyperparathyroidism of kittens on only meat diet, hypovitaminosis D (rickets in growing animals) (3), or hypervitaminosis A (4). There are several types of rickets in growing animals: hypophosphatemic due to loss via kidneys (5) and defects of metabolism of vitamin D—vitamin D-dependent rickets type 1 and type 2 (6), clinical signs are poor growth, soft bones, gait abnormalities, bowing of the legs, and enlarged growth plates, most commonly in the distal limbs (7). Hypervitaminosis A is seen in cats fed excessive amounts of liver and causes multiple bone exostoses, joint laxity, and impingement on nerves causing spinal cord or peripheral nerve and plexus disorders (4). Osteogenesis imperfecta is an inherited bone disease characterized by poor mineralization and excessive bone fragility. Clinically, the disease mimics nutritional secondary hyperparathyroidism (3). Inherited storage diseases, such as mucopolysaccharidoses, can cause long bone and/or vertebral column malformations. Cats with mucopolysaccharidosis present at less than a year of age with abnormal facial features, an abnormal gait, diffuse neurological disease, and dwarfism (3). Clues for the presence of mucopolysaccharidosis are seen in leukocytes as metachromic granules on peripheral blood smears (8).

Endocrine causes of dwarfism are much less common and are hypothyroidism, hyposomatotropism, diabetes mellitus, hypoadrenocorticism, and glucocorticoid excess (1). Hypothyroidism is caused by a developmental failure of the thyroid gland or by impaired thyroid hormone production. Clinical signs include disproportionate dwarfism (short body and limbs, big head), delayed tooth eruption, mental dullness, and lethargy (9, 10). Hypoadrenocorticism was described in one dwarf kitten with clinical signs of neurological disorders, with behavioral changes characterized by excessive fear, recurrent episodes of tremors, weakness, severe hypothermia, dull and scruffy fluffy hair coat, dysorexia, and pica (11).

Recently, the gene for feline dwarfism was described. Structure genome change interrupted UDP-glucose 6-dehydrogenase, a gene involved in the biosynthesis of glycosaminoglycans and was not described in human dwarfism yet (12).

Hyposomatotropism is extremely rare in cats, and only two cases have been described in the literature (13, 14). It is caused by lack of growth hormone (GH) production in the pituitary gland. A recessively inherited disorder has been described in dogs and is seen as a mutation in the LHX3 gene in German shepherd dogs, Saarloos Wolfdogs and Czechoslovakian Wolfdogs (15, 16). Such changes have not yet been detected in cats. In hyposomatotropism, the impaired production of GH in the pituitary gland leads to a secondary deficiency in insulin-like growth factor-1 (IGF-I), which is produced in the liver. The consequence is proportional dwarfism in young cats and may be associated with a wide range of other clinical manifestations such as delayed closure of growth plates, delayed dental eruption, muscle atrophy, retention of secondary hair, lack of primary guard hairs, bilateral symmetrical alopecia, scaling, pyoderma, thin or hyperpigmented skin and testicular atrophy (1, 2, 14), bilateral corneal opacity (13), and seizures due to hypoglycemia (14). Animals are usually presented at 3 to 5 months of age with retardation of growth or skin/coat abnormalities (2, 13, 14, 17).

The diagnostic approach to a dwarf kitten include hematology, serum biochemistry, and urinalysis. Results can aid in identifying renal or liver disease, diabetes mellitus, or others. These tests are usually unremarkable in feline pituitary dwarfism (1). In dogs, azotemia, proteinuria and low specific gravity was observed due to abnormal glomerular development and impaired renal function (2). In one cat, hypoglycemia was described at presentation (14). Fasting hypoglycemia was described in children too (18). Diagnostic evaluation of the skeleton might be needed to evaluate the presence of delayed epiphyseal closure in long bones. Abdominal ultrasound is indicated to exclude other differential diagnoses, such as liver or renal disease (2, 19). Advanced imaging techniques, such as magnetic resonance imaging (MRI) or computed tomography (CT) of the brain, may be useful in diagnosing pituitary dwarfism; in canine cases, the presence of pituitary cysts is usually observed (2, 20).

In terms of endocrine testing, a feline growth hormone (GH) assay has been validated, but is not freely available (21). Moreover, the determination of a single basal GH concentration has little diagnostic value due to its pulsatile secretion pattern. It has been demonstrated in dogs that there is overlap with healthy controls (22). In dogs, stimulation tests using GHRH and other substances are used (2, 20), but none of these tests have been used in cats (1). The determination of insulin-like growth factor (IGF-I) is more reliable because of its non-pulsatile secretion and longer half-life (2, 19). Therefore, the measurement of serum IGF-I concentrations is very useful in determining the level of activity of the somatotropic axis (23). In two diagnosed cats with hyposomatotropism, serum IGF-I was significantly lower [<25 ng/ml (14), <16 ng/ml (13)]. In one reported hypothyroid cat, IGF-I levels were lower too (104 ng/ml, RR 185–525 ng/ml) (24) and normalized with therapy for hypothyroidism. Therefore, evaluation of thyroid gland function is needed to exclude hypothyroidism. The IGF-I evaluation has several limitations since it can be influenced by age, sexual maturity, body size, nutritional status, and illness (1).

The most important effect of GH therapy is to promote growth (height velocity), but GH has also important metabolic effects. Although generalized growth is stimulated, it is not evenly distributed among the protein, lipid, and carbohydrate compartments. In GH-deficient children, hGH therapy results in decreased body fat and increased fat free mass, including muscle and bone. Thus, proper GH secretion probably has major developmental influences on later health risks, including cardiovascular diseases and osteoporosis. The ability of insulin to promote fatty acid synthesis is antagonized by GH. Growth hormone induces a rapid loss of fat due to stimulation of lipolysis and reciprocal antagonism of the lipogenic actions of insulin (25).

In dogs, antibody formation precludes the use of recombinant human GH (26). Administration of porcine GH does not result in antibody formation because the amino acid sequence of porcine GH is identical to that of canine GH (27). The recommended dosage may result in GH excess and side effects, such as DM. Therefore, monitoring of plasma GH and glucose concentrations is recommended three times during each week of treatment (20).

Human growth hormone has been used in GH-deficient children, adolescents, and adult since 1958. Human recombinant GH (rhGH) was approved in 1985 (25). There are a few described side effects observed in therapy of GH deficiency, such as influence of glucose metabolism, since GH physiologically antagonizes insulin effect in glucose and lipid metabolism by stimulating glycogenolysis and lipolysis, and inhibiting glycogenesis and lipogenesis. Thus, treatment with rhGH is assumed to induce insulin resistance (28). Another adverse effect is prepubertal gynecomastia, which is rare and self-limited in treated children (29). Slipped capital femoral epiphysis (SCFE) is defined as a posterior and inferior displacement of the proximal femoral epiphysis on the femoral neck. It occurs more frequently in periods of rapid height gain (30). Children with GH deficiency are more prone to the development of SCFE especially when treated with rhGH. Benign intracranial hypertension (BIH) is the result of the physiological antidiuretic effect of GH and is more evident in patients that cannot support decrease in glomerular filtration rate. Normal children exhibit a mild transitory elevation in plasma renin activity and aldosterone, and rarely develop BIH (31). Malignancies could be another adverse effect as both GH and IGF-I have mitogenic and antiapoptotic properties, and there has always been a concern that rhGH might induce tumorigenesis (32).

This case is unique because it first reported about human recombinant growth hormone therapy in a cat. Moreover, we described the side effects published in human literature.

## Case History

**Figure 1:**
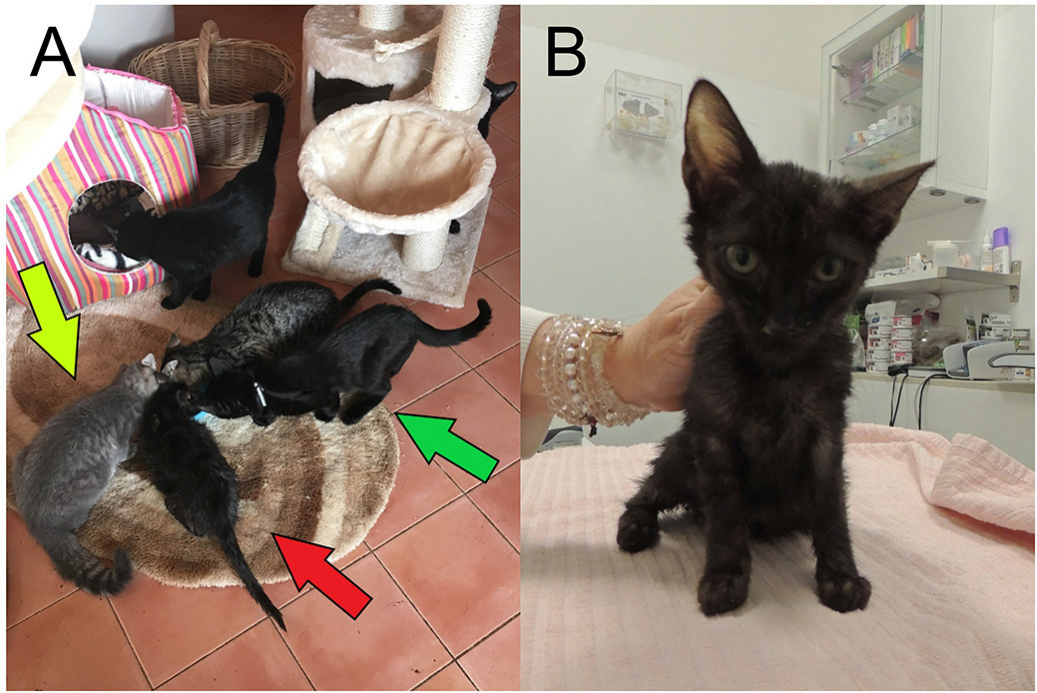
**(A)** Patient at age 6 months (red arrow), two of his litter mates (green arrow), and another cat of the same age (yellow arrow). **(B)** Patient at age 6 months in detail.

A 6-month-old, domestic shorthair tomcat was presented with failure to grow, ocular and nasal discharge, and *Ascaris* infestation. He was half the size of his litter mates (Figure 1). Clinical examination showed 3/9 body condition score, normal corneal opacity, pink moist mucous membranes, poor muscle development, and a body weight of 790 g. According to the literature, the weight of a domestic cat should be ~2.2 kg at this age (33). His fur was dull, and only an undercoat was present, primary guard hairs were missing and the skin was very thin and fragile. Mild alopecia was noted on the ventral neck and on his shoulders. A mild purulent ocular and nasal discharge was present. Both ear canals were filled with thick dark wax, and *Otodectes cynotis* mites were present. All teeth were deciduous. His dwarfism was proportionate, and his legs were proportionate to the rest of the body. Clinical examination was otherwise unremarkable.

Serum biochemistry showed high SDMA (24 μg/dl, RR up to 14, Table 1), slightly elevated AST (56U/L, RR up to 48, Table 1), and T4 was low normal (tT4 17 nmol/L, RR 10–60, Table 1). Calcium level was normal, and phosphorus level was slightly elevated (Table 1), but it is normal in growing animals (34). Furthermore, canine TSH was measured and was unmeasurably low (up to 0.03 ng/ml). Insulin-like growth factor was also unmeasurably low (up to 15 ng/ml). Hematology showed mild non-regenerative anemia (RBC 5.98 × 10^12^/L, RR 6.54–12; HGB 9.6 g/dl, RR 9.8–16.2, HCT 30.5%, RR 30.3–52.3, Table 2) and severe leukocytosis (WBC 49.3 × 10^9^/L, RR 2.87–17.02, Table 2) with neutrophilia, left shift, and monocytosis. Blood smear confirmed leukocytosis with the presence of band neutrophil, mild toxic changes in the cytoplasm of neutrophils, mildly reactive lymphocytes, and platelet clots. Urine examination was unremarkable, UP:UC ratio was 0.06, urine culture negative, and USG 1.053. Urine sediment exam revealed five casts, a few epithelia, no crystals, erythrocytes, leukocytes, or bacteria. A fecal analysis, including flotation and ELISA examination for the presence of *Giardia* and *Cryptosporidium* spp., was negative.

**Table 1:** Blood biochemistry results.

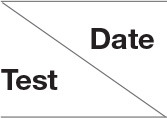	**12/01** **2019**	**01/31** **2020**	**02/16** **2020**	**03/01** **2020**	**05/14** **2020**	**09/26** **2020**	**Reference range**	**Unit**
Canine TSH	<0.03		0.03				<0.5	ng/ml
IGF-I	<15.0		412		323	453	50–665	ng/ml
GLU	5.51	5.63	5.82	5.61			4.11–8.84	mmol/L
SDMA	24	8	11				0–14	μg/dl
CREA	55	147	103	75			71–212	μmol/L
UREA	8.8	10.6	9.1	7.4			5.7–12.9	mmol/L
BUN/CREA	40	18	22	25				
PHOS		2.74	2.59	2.76			1.00–2.42	mmol/L
CA		2.52	2.52	2.62			1.95–2.83	mmol/L
TP	69	69	68	72			57–89	g/L
ALB	26	26	26	27			22–40	g/L
GLOB	43	43	42	44			28–51	g/L
ALB/GLOB	0.6	0.6	0.6	0.6				
ALT	86	155	416	158			12–130	U/L
AST	56	85	156	52			0–48	U/L
ALKP	95	173	174	215			14–111	U/L
GGT		3	0	0			0–4	U/L
TBIL		<2	3	3			0–15	μmol/L
CHOL		2.40	2.55	3.07			1.68–5.81	mmol/L
Na			157	157			150–165	mmol/L
K			3.9	3.9			3.5–5.8	mmol/L
Na/K			41	40				
Cl			122	115			112–129	mmol/L
OsmCalc			314	312				mmol/kg
TT4	17		25				10–60	nmol/L

**Table 2:** Blood hematology results.

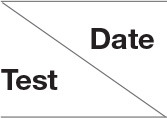	**12/01** **2019**	**01/31** **2020**	**Reference range**	**Unit**
RBC	5.98	7.98	6.54–12.20	x10^12^/L
HCT	30.5	37.7	30.3–52.3	%
HGB	9.6	12.3	9.8–16.2	g/dl
MCV	51.0	47.2	35.9–53.1	fl
MCH	16.1	15.4	11.7–17.3	pg
MCHC	31.5	32.6	28.1–35.8	g/dl
RDW	22.8	24.2	15.0–27.0	%
%RETIC	0.3	0.1		%
RETIC	17.9	10.4	3.0–50.0	K/μl
RETIC-HGB	16.5	19.2	13.2–20.8	pg
WBC	49.30	18.88	2.87–17.02	x10^9^/L
%NEU	86.2	69.7		%
%LYM	7.5	23.0		%
%MONO	5.9	4.9		%
%EOS	0.2	2.0		%
%BASO	0.2	0.4		%
NEU	42.53	13.17	2.30–10.29	x10^9^/L
LYM	3.69	4.35	0.92–6.68	x10^9^/L
MONO	2.29	0.92	0.05–0.67	x10^9^/L
EOS	0.10	0.37	0.17–1.57	x10^9^/L
BASO	0.08	0.07	0.01–0.26	x10^9^/L
PLT	424	311	151–600	K/μl
MPV	16.9	17.6	11.4–21.6	fl
PCT	0.72	0.55	0.17–0.86	%

**Figure 2:**
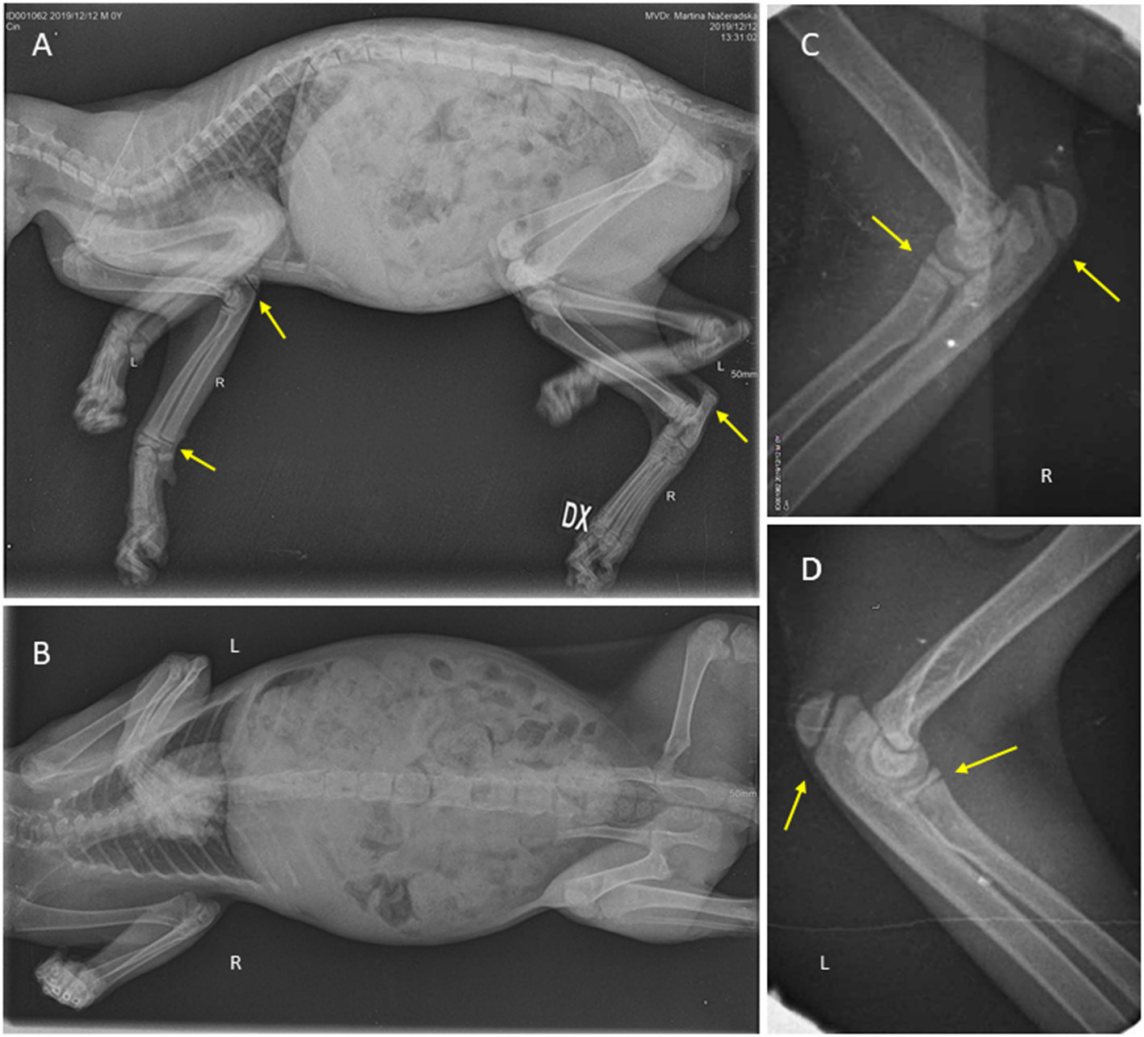
Patient at the beginning of the therapy; whole-body X-ray show normal mineralization of the bones, and no fractures present. **(A)** Lateral position with marked normal growth plates (yellow arrows). **(B)** Ventrodorsal position. Elbows in detail. **(C)** Lateral position of the right elbow. **(D)** Lateral position of the left elbow, both with normal growth plates (yellow arrows).

Eye, throat, and nose swabs were performed for PCR for *Mycoplasma, Ureaplasma, Chlamydia*, FHV-1, and calicivirus. The results came back very highly positive for *Mycoplasma* spp. (10^7^/ml), highly positive for FHV-1 (10^4^/ml), and mildly positive for *Ureaplasma* (10^3^/ml). X-rays (Figure 2) showed the front legs to be similar to a 16-week-old cat and the hind legs similar to a 12-week-old cat according to the literature (35). Growth plate closure was slightly delayed, but the growth plates were not deformed. Ultrasonography of the abdomen did not reveal any pathology; liver and kidneys were of normal echogenicity and size.

Differential diagnoses included pituitary dwarfism, severe infection, ascarid infestation, and portosystemic shunt (less likely).

## Diagnostic Assessment

Therapy with peroral antibiotics [amoxicillin–clavulanate 25 mg/kg BID (Noroclav, Norbrook, UK)], spiramycin (75,000 IU/kg), and metronidazole (12.5 mg/kg) orally once a day (fixed dose combination; Stomorgyl, Merial, France) was started and given for 10 days. The *Ascaris* and *Otodectes* infestation was treated with a spot-on preparation (selamectin, Stronghold, Zoetis, USA). Antibiotic treatment was changed once PCR results were received 10 days later. Pradofloxacine 3 mg/kg SID (Veraflox, Bayer, Germany) was given perorally for 3 weeks, but the therapy was not sufficient, as both *Ureaplasma* and *Mycoplasma* were still present at this point, and therapy was changed to doxycycline 10 mg/kg SID (compounded syrup from pharmacy) for 4 weeks. One month later, the cat was clinically cured, and PCR came back negative. Treatment for FHV-1 included two 1-week courses of famciclovir 62.5 mg *pro toto* BID (Famvir, Novartis, Switzerland) and was later PCR negative.

**Figure 3:**
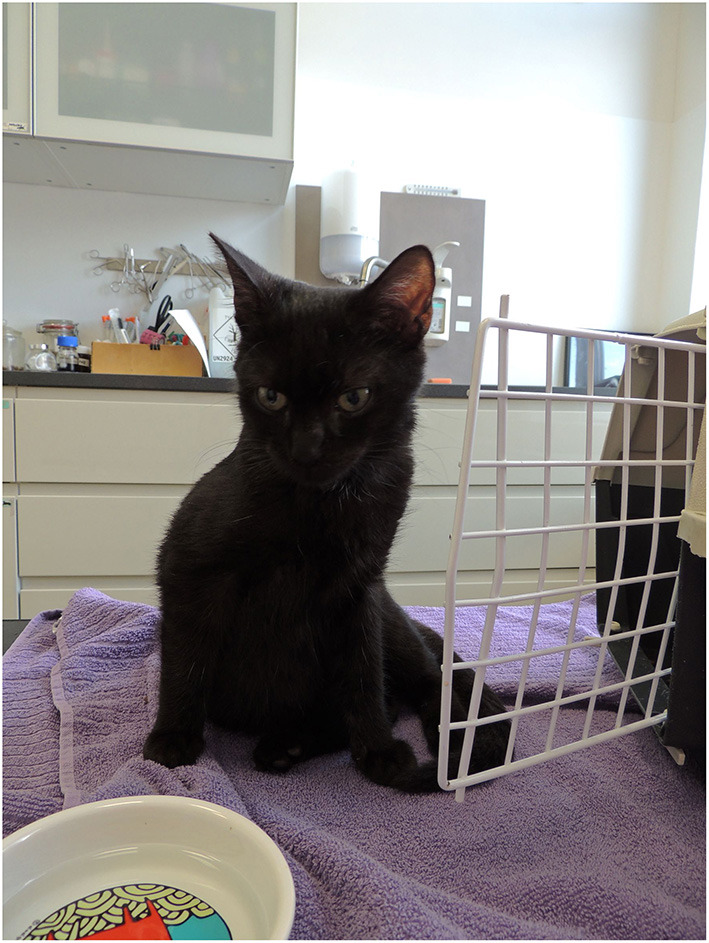
Patient at age of 8 months, weight 2.7 kg.

After receiving the results of IGF-I assay, therapy with human recombinant growth hormone subcutaneously (Humatrope 36 IU (12 mg) 1 × 3.15-ml injection, Eli Lilly and Company, USA) was started. The cat received 1.6 mg of human recombinant growth hormone *pro toto* twice weekly for 9 weeks. The dose was extrapolated from the human growth hormone-deficient pediatric patient dose of 0.24–0.37 mg/kg/week (36). Since the metabolism of hormones is much faster in cats than in humans (37, 38), the dose was 10 times higher than the maximum dose recommended in humans. During therapy, liver enzymes got elevated, and treatment was discontinued. After 7 weeks, the liver enzymes were mildly elevated (ALT 155 U/L, RR 12–130, AST 85 U/L, RR 0–48 and ALP 173 U/L, RR 14–111, GGT was normal, Table 1), and after 9 weeks, the enzymes were much higher (ALT 416 U/L, AST 156 U/L, ALP 174 U/L, GGT was normal, Table 1). Therapy with growth hormone was discontinued at 8 months of age (Figure 3). Within 2 weeks of growth hormone treatment cessation, liver enzymes decreased almost back to normal (ALT 158 U/L, AST 52 U/L, Table 1). IGF-I was measured at discontinuation of therapy, and it was within the normal reference range (412 ng/ml, RR 50–665, Table 1). Normal IGF-1 was present 3 months after discontinuation of therapy (323 ng/ml) with human recombinant growth hormone as well as 6 months later (453 ng/ml).

Within the first 2 weeks of therapy, his permanent teeth started breaking through. Within a month, his fur changed, and primary guard hairs appeared, and the kitten became almost the same size of his littermates and got adopted by first author.

## Discussion

Malnutrition as a cause of dwarfism was less likely, since the kitten was first at the bowl, seen eating many times a day, and he was ravenous while eating and still is. That is maybe the reason why he never developed hypoglycemia described in one case of the cat with hyposomatotropism (14). Malnutrition in a small cat with a very good appetite being fed an adequate diet may be secondary to maldigestion or malabsorption (19), and since he had soft stool and *Ascaris* manifestation, this could have contributed to his small stature. He had severe leukocytosis with a left shift, which points to severe infection contributing to his dwarfism as described in the literature (1).

Mucopolysaccharidosis was ruled out, as metachromic granules should be seen in leukocytes on peripheral blood smear according to the literature (8). Moreover, he had no ocular manifestations as described in the literature (13).

Portosystemic shunt was one of the possible causes, but pre- and post-prandial bile acid screening was not performed as the kitten was recovering well on current therapy. Hepatic and renal disease were excluded via ultrasound, blood, and urine exam on first visit. He did not have any heart murmur, so heart disease was unlikely.

Skeletal x-rays revealed no signs of systemic diseases that can cause abnormal skeletal development except delayed proportional growth. No exostoses or deformations of the cartilage nor growth plates were seen. No demineralization or widening of growth plates was seen, so that discrepancy in minerals and vitamin D metabolism was not suspected. His calcium and phosphorus levels were also normal according to his age (34).

This kitten was severely underweight, which makes maldigestion or severe disease more likely, compared with endocrinopathies, where affected animals tend to be more overweight (8). Infection and inflammation were most likely contributing to the dwarfism. In conjunction with maldigestion, the cat suffered from severe infection, which contributed to the low IGF-I, and this explains why his IGF levels were normal even after withdrawal of growth hormone supplementation.

Congenital hypothyroidism was very unlikely since the dwarfism was not disproportionate, and the kitten was not mentally dull (10). The level of the thyroid hormone was in the low reference range at the beginning and normal while on therapy. His TSH was unmeasurably low at the beginning and later normal. There may be a connection between growth hormone treatment and thyroid function as described in one kitten treated for hypothyroidism (24). Congenital hypoadrenocorticism was unlikely since his minerals were normal (Table 1) compared with one described case (11), and he reacted well to the given therapy.

Since MRI or CT was not performed, hyposomatotropism could not be excluded as a cause of dwarfism in this cat. On x-ray, delayed closure of growth plates was seen, and he had delayed dental eruption, muscle atrophy, retention of secondary hair, lack of primary guard hairs, symmetrical alopecia on his neck, and thin skin, which is compatible with hyposomatotropism (1, 2, 14). A recently described genome change as a cause of dwarfism could not be excluded, since the genome was not tested (12).

Hormone metabolism in the cat differs from that in humans. Absorption is similar, but the pharmacokinetics are different (37). Hormones are metabolized mainly via sulfatation, and this way of metabolism is much faster in cats than in dogs and humans (38). This is why the dose used was 10 times higher than in human medicine. Monitoring of IGF-I is recommended (1), and that is what we did. Interestingly, the normal IGF-I level remained stable even after discontinuation of the therapy with human recombinant hormone.

In human medicine, therapy is usually discontinued after the end of bone growth (39). In this case, therapy was discontinued earlier because of an elevation of liver enzymes. Some human adults still need GH therapy even in adulthood (39). In this feline case, this was not necessary. Self-limiting liver enzyme elevation was described in one study in human medicine in 3.8% of children on growth hormone therapy, and further investigation did not reveal any other cause. Liver enzymes normalized spontaneously within 3 to 6 months, without stopping the therapy (40). It seems like the same happened here: self-limiting liver enzyme elevation, which normalized with discontinuation of therapy.

In humans, the main side effect of therapy with human recombinant growth hormone is high glucose or diabetes mellitus because of its influence on glucose metabolism, none of which was observed in our case. Blood glucose was normal at the beginning of the therapy, 7 weeks later, and at the time of discontinuation of therapy. Another side effect described in humans is lower thyroid hormone levels, which was also not observed in our case; the thyroid hormone level was higher (25 nmol/L) than at the beginning. This might suggest a connection between levels of IGF-I and thyroid hormones because therapy of congenital hypothyroidism leads to an increase in the level of IGF-1, which was previously low, in one described case (24). Further side effects, such as SCFE, were not observed on x-ray. Clinical signs of BIH, such as headache, are hard to observe in cats, but vomiting was not observed.

Given all the facts, we can conclude that the cat suffered from dwarfism, which could be caused by severe infection and/or maldigestion. Pituitary dwarfism cannot be excluded either, since no advanced diagnostics were performed, and the IGF-1 was unmeasurably low at the beginning of the therapy.

## Data Availability Statement

The original contributions presented in the study are included in the article/supplementary material, further inquiries can be directed to the corresponding author.

## Ethics Statement

Ethical review and approval was not required for the animal study because this was case report, cat owned of one of the authors. Written informed consent was obtained from the owners for the participation of their animal in this study.

## Author Contributions

MN created the concept of the work. MN, KNH, and MF collected the data and prepared the manuscript. MN and MF did the final check. All authors contributed to the article and approved the submitted version.

## Conflict of Interest

The authors declare that the research was conducted in the absence of any commercial or financial relationships that could be construed as a potential conflict of interest.

## Publisher's Note

All claims expressed in this article are solely those of the authors and do not necessarily represent those of their affiliated organizations, or those of the publisher, the editors and the reviewers. Any product that may be evaluated in this article, or claim that may be made by its manufacturer, is not guaranteed or endorsed by the publisher.
